# Effects on postgraduate-year-I residents of simulation-based learning compared to traditional lecture-style education led by postgraduate-year-II residents: a pilot study

**DOI:** 10.1186/s12909-019-1509-y

**Published:** 2019-03-20

**Authors:** Akira Yamamoto, Mikako Obika, Yasuhiro Mandai, Taku Murakami, Tomoko Miyoshi, Hideo Ino, Hitomi Kataoka, Fumio Otsuka

**Affiliations:** 1 Department of General Medicine, Okayama University Graduate School of Medicine, Dentistry and Pharmaceutical Sciences, 2-5-1 Shikata-cho, Kita-ku, Okayama, 700-8558 Japan; 2 Center for Education in Medicine and Health Sciences, Okayama University Graduate School of Medicine, Dentistry and Pharmaceutical Sciences, 2-5-1 Shikata-cho, Kita-ku, Okayama, 700-8558 Japan; 3 Department of Primary Care and Medical Education, Okayama University Graduate School of Medicine, Dentistry, and Pharmaceutical Sciences, 2-5-1 Shikata-cho, Kita-ku, Okayama, 700-8558 Japan

**Keywords:** Simulation-based learning, Peer-assisted learning, Lecture, Postgraduate education, Junior residents

## Abstract

**Background:**

Simulation-based learning plays an important role in contemporary medical education, although there are problems providing tutors. Peer-assisted learning has begun being formally adopted in medical education. Although it is considered useful for simulation-based learning, its effectiveness remains unclear. This study was designed to compare the effect of simulation-based learning with that of traditional lectures conducted by postgraduate-year (PGY)-II residents on PGY-I residents.

**Methods:**

This study was conducted at Okayama University Hospital over three years, for one week each year, before residents entered clinical practice. The study enrolled 76 PGY-I residents, who were randomized into two groups: simulation and lecture groups. PGY-II residents volunteered to conduct simulations and lectures. Knowledge evaluation was performed using pre- and post-tests, and self-evaluation of competence and behaviour-change and program evaluations were conducted using questionnaires.

**Results:**

In both groups, knowledge test scores were found to improve significantly, and the score difference between pre- and post-tests in both the groups was not significant. Self-evaluation of competence and behaviour-change was found to be higher in the simulation group than the lecture group. The trainees in the simulation grou*p* valued the program and the PGY-II residents as teaching staff more than those in the lecture group.

**Conclusions:**

The combination of simulation-based learning and peer-assisted learning led by PGY-II residents is potentially more effective in improving the postgraduate education of PGY-I residents than the combination of lecture and peer-assisted learning.

## Background

Previous medical training using the philosophy of ‘see one, do one, teach one’ posed inherent risks to both patients and trainees. Simulation-based learning can provide a risk-free environment where trainees are permitted to make mistakes, which will reduce the occurrence of errors at clinical sites [1, 2]. A meta-analysis showed that simulation-based learning was more effective than traditional education for trainees to acquire medical technical skills [3]. However, the effectiveness of simulation-based learning for the acquisition of initial case management skills, which junior residents are required to learn, has not yet been confirmed. This study aims to examine this research gap.

On the practical side of postgraduate medical education in Japan, postgraduate-year-I (PGY-I) residents often receive formal and informal ‘near’ peer-assisted learning (PAL) from PGY-II residents. PAL is defined as follows: ‘trainees from similar social groupings who are not professional teachers helping each other to learn and learning themselves by teaching’. [4] To the best of our knowledge, there are no studies that report the effects of simulation-based learning conducted by PGY-II residents for PGY-I residents, although many studies have revealed that PAL and simulation-based learning have educational benefits [5, 6].

Therefore, we offered a simulation-based learning and traditional lecture course conducted by PGY-II residents for PGY-I residents. We evaluated this program using three measures (knowledge evaluation, self-evaluation of competence and behaviour-change, and program evaluation) and compared the benefits of simulation-based learning with those of traditional lectures.

## Methods

**Fig. 1:**
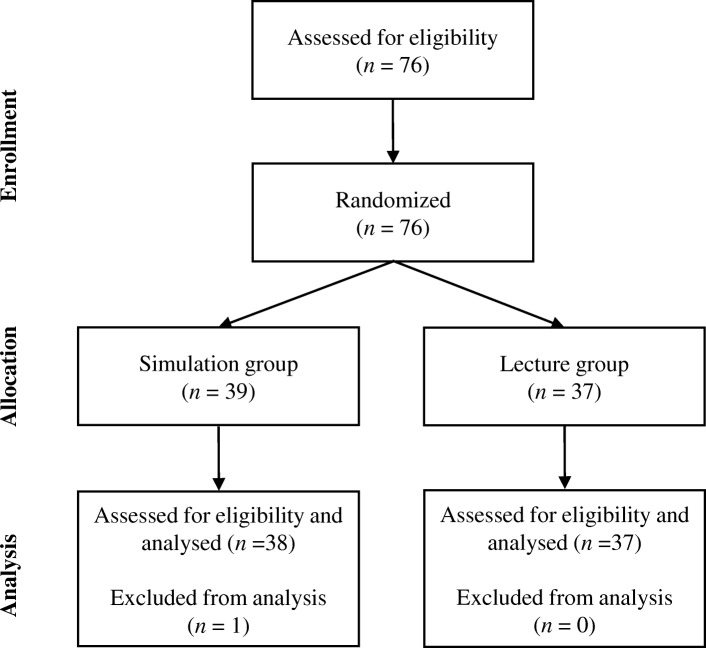
Selection and categorisation of participants in the study

This interventional education study was conducted at Okayama University Hospital for one week each year for three years on PGY-I residents before they began clinical practice. During the specified week, new employee training was provided to teach the residents what they needed to know about working in the hospital, such as how to use electronic medical records. We randomized the residents by the order of their names in the Japanese syllabary and divided them into two groups (Fig. 1): simulation-based learning (simulation group, *n* = 38) and traditional lecture style (lecture group, *n* = 37). One resident was excluded because of insufficient data. Each year, we evaluated the PGY-I residents who had agreed to participate.

To develop case scenarios and goals, in the year prior to conducting this study, we asked PGY-I residents about the type of cases they should experience in a PGY-I internship. Based on their responses, we conducted an alpha test comprising simulations of candidate cases and evaluated the difficulty, validity, accuracy, and effectiveness of the cases. Following the alpha test, we conducted a beta test consisting of experimental simulations with the residents to evaluate and calibrate cases. Finally, we determined three case scenarios (acute myocardial infarction, multiple injuries, and aspiration pneumonia) that were considered feasible and effective for PGY-I residents (Table 1). All teaching materials were closely examined by experts.

**Table 1:** Details of scenarios

	Simulation group	Lecture group
	(*n* = 38)	(*n* = 37)
Cases and goals	Case 1. An outpatient with acute myocardial infarction	
	Understanding basic physical examination methods of walk-in patients.	
	Understanding the initial evaluation and examination of patients with chest pain.	
	Understanding differential diagnosis of fatal chest pain.	
	Understanding acute myocardial infarction.	
	Case 2. A patient with multiple injury coming by an ambulance	
	Understanding the initial evaluation and treatment of high energy trauma.	
	Understanding imaging findings of intraabdominal bleeding and multiple fractures.	
	Case 3. An inpatient with pneumonia	
	Understanding the initial evaluation and treatment at the time of sudden change to a fetal disease of a hospitalized patient.	
	Understanding the method of report to an advanced doctor.	
	Understanding pneumonia.	
total time of each case	one hour	one hour
detail of time coarse (minutes)	explanation of equipment (5)	lecture (60)
	case presentation (1) †	
	team discussion (3) †	
	simulation training (5) †	
	debriefing (8) †	
	†Repeating 3 times	
Number of each group	12–14††	11–14
	††divided to two groups in this group	
Teaching staff	Volunteer postgraduate-year-II residents	Volunteer postgraduate-year-II residents

Each year, volunteer PGY-II residents conducted simulations and lectures as teaching staff. In the simulation group, PGY-I residents were divided into two groups by name. A few minutes were spent explaining the equipment, following which case presentation, team discussion, simulation training, and debriefing took 1, 3, 5, and 8 min, respectively. Since debriefing is considered the most important part of a simulation, we allocated more time to it, and the teaching staff received debriefing education before participating in the program [7]. They repeated the scenario, excluding the explanation of the equipment, three times while changing the roles of PGY-I residents as a doctor, a nurse, and a secretary writing down their orders or the treatment for the debriefing. Each scenario took a total of one hour. They were provided simulation tools such as *SimMan 3G*, a monitor, an emergency cart with medications, an intravenous drip, a cardiac defibrillator, an intubation instrument, and a white board to record the scenario’s progress. The teaching staff presented the results of blood, X-ray, CT scan, electro-cardiogram, and ultrasound sonography tests when requested by trainees. The lecture group received a one-hour, one-way communication lecture based on the same case and goals that were used in simulation-based learning. Both groups had three sessions per week. Every year, after finishing the program, all residents were given the opportunity to participate in simulation-based learning and lectures.

**Fig. 2:**
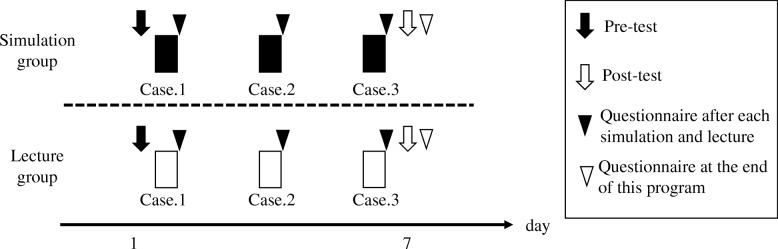
Outline of the study. Black rectangle: simulation for 1 h, Case.1 to Case.3. White rectangle: lecture for 1 h, Case.1 to Case.3. Black arrow: pre-test. White arrow: post-test and questionnaire. Black arrowhead: questionnaire administered immediately after each simulation and lecture. White arrowhead: questionnaire administered at the end of the program

For knowledge evaluation (Fig. 2), we prepared a pre- and a post-test, including 24 multiple-choice and 28 free-response questions based on the three scenarios (adjusted to a total of 0–100 points). For both groups, we administered a questionnaire immediately after each simulation and lecture for the self-evaluation of competence and program evaluation. Regarding the self-evaluation of competence, the questionnaire asked the degree to which the participants’ knowledge, judgement, and skills had improved and their degree of confidence in seeing a patient (1–10 points each; 4–40 points in total). For program evaluation, the questionnaire asked about the difficulty of the case and meaningfulness of the program (1–10 points) (Table 2). At the end of the program, a questionnaire was used in all groups for the self-evaluation of behaviour-change and program evaluation (Table 3). Regarding the self-evaluation of behaviour-change, the questionnaire asked about the residents’ self-learning time after each training session (1–5 points) and the degree of behaviour-change. For program evaluation, questions were asked about the degree of stress that resulted from this program, residents’ expectations that this program would relieve stress in clinical situations, the appropriateness of the program’s timing, the competency of teaching staff, and the residents’ recommendation whether this program should be continued (1–10 points). The questionnaires included adequate space for free responses about the program and teaching staff, as well. The questionnaires were created after literature review and expert validation [8, 9].

**Table 2:**
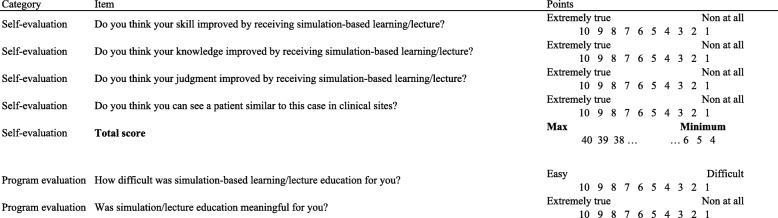
Questionnaire administered immediately after each simulation/lecture (depicted using black arrowheads in Fig. 2)

**Table 3:**
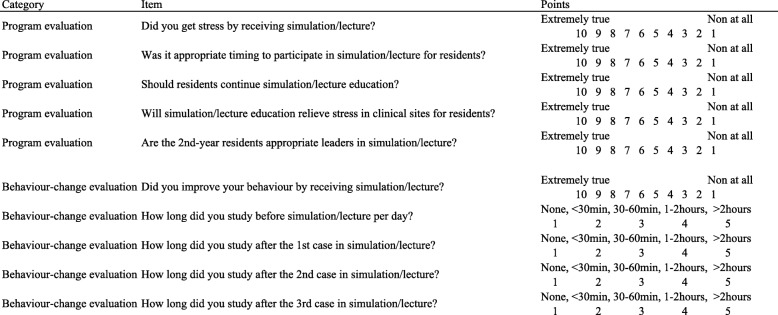
Questionnaire administered at the end of the program (depicted using white arrowheads in Fig. 2)

The statistical analysis of the population was conducted using a chi-squared test. Further, Student’s t-test was used to compare knowledge test scores and the total scores of the questionnaires after each simulation and lecture. In addition, a paired t-test was used to determine the improvement in knowledge test scores and self-evaluations after cases. For other data that required nonparametric tests, the Mann-Whitney test was used. In addition, we controlled the family-wise error rate using the Benjamini-Hochberg’s method for the adjustment of multiple comparisons [10]. The statistical analyses were performed using SPSS v.24.0 software for Windows.

This study was approved by the ethics committee of the Okayama Medical School.

## Results

### Population

There were no significant differences in the baseline demographic data of the two groups in the following characteristics: gender, experience in simulation-based learning as a trainee or as teaching staff, motivation to become teaching staff for simulation education, being a graduate of the Okayama Medical School, and self-study time each day (*p* > 0.05) (Table 4).

**Table 4:** Baseline demographic data

Resident characteristics					
	Simulation		Lecture		*p value*
Male: Number of affirmation (%)	21	(55)	22	(60)	0.71
Have you ever had simulation-based learning in medical school?: Number of affirmation (%)	32	(84)	27	(73)	0.24
Have you ever been a tutor of simulation-based learning?: Number of affirmation (%)	7	(18)	6	(16)	0.84
Would you like to instruct simulation training?: Number of affirmation (%)	22	(18)	19	(51)	0.57
Did you belong to a study group or training club in medical school?: Number of affirmation (%)	15	(40)	12	(32)	0.53
Did you graduate from Okayama medical school?: Number of affirmation (%)	19	(50)	20	(54)	0.73
How long do you conventionally study everyday?: Median points† (interquartile range) [points]	1	(1)	1	(1)	0.32

†None, 1; < 30 min, 2; 30-60 min, 3; 1-2 h, 4; > 2 h, 5

### Knowledge evaluation

The pre-test scores of the two groups were not statistically different (mean score of simulation group, 52.3; mean score of lecture group, 54.5; 95% confidence interval [CI], − 9.3 to 5; *p* value, 0.50). A comparison of their pre-tests and post-tests revealed that both the groups improved their scores (*p* < 0.001) (Table 5). Further, no difference was detected in score improvement between the groups (*p* > 0.05).

**Table 5:** Knowledge evaluation using pre- and post-test scores (points)

	Pre-test		Post-test			
	Mean	SD	Mean	SD	difference	*p value*
Simulation group	52.3	12.3	69.6	9.3	17.3	<0.001**
Lecture group	54.5	13.9	70.9	11.1	16.7	<0.001**

*p* < 0.05, adjusted for multiple tests

**p ≤ 0.001, adjusted for multiple tests

### Self-evaluation of competence and behaviour-change

The total self-evaluation of competence scores after each scenario in the simulation group were higher than those in the lecture group (Case, simulation group mean points (SD) - lecture group mean points (SD), *p* value: Case.1, 31.1 (4.3) – 28.2 (6.8), 0.04; Case.2, 32.2 (4.7) – 28.9 (5.4), 0.02; Case.3, 33.2 (4.3) – 29.9 (6.5), 0.02) (Table 6).

**Table 6:** Self-evaluation using questionnaire (1–10 points)

Item	Simulation		Lecture		
	Mean	SD	Mean	SD	*p value*
Total score of self-evaluation					
Case.1	31.1	4.3	28.2	6.8	0.04*
Case.2	32.2	4.7	28.9	5.4	0.02*
Case.3	33.2	4.3	29.9	6.5	0.02*

SD, standard deviation

*p < 0.05, adjusted for multiple tests

Following simulation-based learning and lectures, the residents in the simulation group thought that their behaviour improved more than the behaviour of those in the lecture group (simulation group median points interquartile range (IQR), lecture group median points (IQR), *p* value: 9 (1)– 7.5 (2.75), < 0.001, respectively) (Table 7). Regarding specific behaviour changes, the simulation group spent more time on self-learning than the lecture group, with a significant difference after each scenario (Case, simulation group median points (IQR) - lecture group median points (IQR), *p* value: Case.1, 2 (0) - 2 (1), 0.001; Case.2, 2 (1.5) - 1 (1), 0.02; Case.3, 2 (2) - 1 (1), 0.02).

**Table 7:** Behaviour-change evaluation

Item	Simulation		Lecture		
	Median	IQR	Median	IQR	*p value*
Did you improve your behaviour by receiving simulation/lecture?	9	1	7.5	2.75	< 0.001**
Self-learning time after each case.					
Case.1	2	0	2	1	0.01*
Case.2	2	1.5	1	1	0.03*
Case.3	2	2	1	1	0.02*

Questionnaire of behaviour change (1–10 points) and self-learning time (1–5 points)

IQR, interquartile range

*p* < 0.05, adjusted for multiple tests

**p ≤ 0.001, adjusted for multiple tests

### Program evaluation

The residents in the simulation group felt that Case.1 was more difficult than did those in the lecture group (simulation group median points (IQR) - lecture group median points (IQR), *p* value: 5 (2) - 6 (2), 0.01) (Table 8). In a free response questionnaire about this program (Table 9), a few residents in the simulation group felt nervous about Case.1. However, after Case.2, there were no differences in the scores of difficulty (*p* > 0.05) or in stress from each educational intervention (*p* > 0.05), and no comments were made in the free response questionnaire about being nervous. Further, there were no differences in responses regarding the appropriate timing of the simulation and lecture (*p* > 0.05).

**Table 8:** Program evaluation by questionnaire (1–10 points)

Item	Simulation		Lecture		
	Median	IQR	Median	IQR	*p value*
How difficult was simulation-based learning/lecture education for you?					
Case.1	5	2	6	2	0.01*
Case.2	5	2	6	1	0.27
Case.3	5	1	6	2	0.09
Was simulation/lecture education meaningful for you?					
Case.1	9.5	1	8	2	0.01*
Case.2	10	1	9	2	0.01*
Case.3	9.5	2	9	2	0.04*
Did you get stress by receiving simulation/lecture?	4	3	4	3.75	0.17
Is it appropriate timing to participate in simulation/lecture for residents?	9	1.5	8	3	0.06
Should residents continue simulation/lecture education?	10	1	8	2	0.001**
Will simulation/lecture education relieve stress in clinical sites for residents?	9	1.5	8	2	0.001**
Are PGY-II residents appropriate teaching staffs in simulation/lecture?	10	1	9	2	0.02*

IQR, interquartile range

*p < 0.05, adjusted for multiple tests

**p ≤ 0.001, adjusted for multiple tests

**Table 9:** Program evaluation by free description questionnaire of the program

Simulation group	Lecture group
Context	Context
I thank to teaching staffs.	I learned a lot from this case.
I was motivated to learn more.	This case was easy for me to understand.
I learned a lot from this training.	I thank to teaching staffs.
This training was meaningful for me.	I understood the lack of my medical knowledge and skills.
I understood the lack of my medical knowledge and skills.	I was motivated to learn more.
I was able to assume the actual clinical site.	The teaching staffs are good at teaching.
This case was difficult for me to understand.	This case was difficult for me to understand.
I enjoyed this training.	
I felt my growth from Case.1 in Case. 2.	
I took this training using what I learned in the previous training.	
I was nervous in this training in Case. 1.	
I learned an importance of this training.	
By receiving this training, I would see outpatients in emergency department at my ease.	

The simulation group felt the training they participated in was more meaningful than did the lecture group (Case, simulation group median points (IQR) - lecture group median points (IQR), *p* value: Case.1, 9.5 (1) - 8 (2), 0.01; Case.2, 10 (1) - 9 (2), 0.01; Case.3, 9.5 (2) - 9 (2), 0.03). Further, those in the simulation group felt more strongly that this training should continue in the future than did the participants in the lecture group (simulation group median points (IQR) - lecture group median points (IQR), *p* value: 10 (1) - 8 (2), 0.001, respectively). In a free response questionnaire about this program, they commented that they were motivated to learn more, learned a lot from the simulation-based training, had meaningful training, and so on.

The simulation group felt that participating in this training would relieve their stress in clinical situations more than did the lecture group (simulation group median points (IQR) - lecture group median points (IQR), *p* value: 9 (1.5) - 8 (2), 0.001, respectively) with comments in the free response portion of the questionnaire that they felt able to assume responsibilities at actual clinical sites.

Further, in the free response questionnaire about the teaching staff, both groups valued PGY-II, and no comments mentioned that the PGY-II residents were not appropriate teaching staff (Table 10). They commented that the reasons PGY-II residents were appropriate teaching staff included teaching them specifically what junior residents should and could do and having no boundaries regarding the questions they could ask. There was a significant difference between the two groups in the questionnaire scoring; the simulation group was more positive about PGY-II residents as appropriate teaching staff than the lecture group (simulation group median points (IQR) - lecture group median points (IQR), *p* value: 10 (1) - 9 (2), 0.02, respectively).

**Table 10:** Program evaluation by free description questionnaire of teaching staff

Simulation group	Lecture group
Context	Context
PGY-II residents were proper teaching staffs, because	PGY-II residents were proper teaching staffs, because
they taught what junior residents should and could do concretely.	they taught us in the same position as us.
they had no boarder to ask a question.	what they taught was easy to understand.
they provided a relaxing environment.	they had no boarder to ask a question.
they knew PGY-I residents’ feelings and sympathized with us.	they taught us based on their experience in PGY-I residents days.
they were good at teaching.	they knew what we did not know and should learn.
they taught us based on their experience in PGY-I residents days.	what they taught was packed with materials we wanted to learn.
what they taught was likely to be practiced soon in clinical sites.	
they taught us kindly and carefully.	
they were passionate.	
they were our goal one year from now.	
they motivated us to learn more.	

## Discussion

Compared to lectures incorporating PAL, simulation-based learning led by PGY-II had remarkable benefits for PGY-I residents from the perspective of the latter’s knowledge acquisition and self-evaluation. Based on these data, we categorized the effects of this study into three themes: knowledge evaluation, self-evaluation of competence and behaviour-change, and program evaluation.

## Knowledge evaluation

In terms of acquiring knowledge, the effect of simulation-based training on PGY-I residents was equivalent to that of lectures. Since the pre- and post-tests were self-derived, our data had limitations; however, these results have already been revealed by previous research [11]. A simulation is a process through which knowledge is translated into reasoned action [2]. In other words, a simulation translates ‘knows’ and ‘knows how’ into ‘shows how’ in the framework for clinical assessment specified by Miller [12]. The simulation group’s improvement in clinical skills was evaluated by themselves and also by the PGY-II residents using checklists evaluating ‘shows how’ when it was detected (data not shown). Therefore, this suggests that simulation-based learning has educational effects on not only acquisition of knowledge but also improvement in ‘shows how’, whereas lectures are limited to the acquisition of knowledge.

## Self-evaluation of competence and behaviour-change

Many studies have reported that simulation-based learning or PAL assists in the learning of medical procedures [5, 13]. Researchers have evaluated the educational effects on trainees using procedure time, checklists, written examinations, and other methods. In this study, we focused more attention on and evaluated trainees’ awareness and behavioural changes, rather than their medical skills themselves. We chose this approach because educational methods that do not promote awareness, called ‘spoon-feeding education’, are not persistently effective for trainees, even if a trainee acquires knowledge or skills.

Self-efficacy affects individual behaviour to achieve goals. In the self-evaluations, it was suggested that simulation-based learning improved trainees’ self-efficacy. Further, the simulation-based learning program was developed to include the concept of self-efficacy. Self-efficacy is known to have a substantial effect on a trainee’s performance, particularly in nursing education [14]. It is derived from four principal sources of information: performance accomplishments, various experiences, verbal persuasion, and physiological states [15]. We provided all these sources in the simulation-based learning program. Trainees achieved their own successful experience (performance accomplishments) and saw what the others did in the same scenario (various experiences). In the debriefing, they could learn from their mutual reviews and the facilitator (verbal persuasion). In addition, a facilitator asked about their feelings when the training was done to objectively make the trainees aware of their feelings and revise them through mutual reviews (physiological states). Further, receiving simulation-based training while being observed by their peers seemed to differ from being trained by specialists whose positions were completely different from the perspectives of affinity and tension. The PAL conducted by PGY-II residents created a less stressful and more relaxed educational environment, as they commented in the free response questionnaire (physiological states). We detected that their self-efficacy gradually improved in the simulation group. We used high-fidelity patient simulators, such as *Simman* and real equipment, since previous research has shown that self-efficacy improves after the use of high-fidelity patient simulator scenarios [14]. In contrast, lecture-style education has only a few of these elements. Therefore, we concluded that PGY-II residents were better teaching staff in simulation-based learning than in lectures from the perspective of self-efficacy.

The time spent on self-learning by residents increased, which, as a rule of thumb in Japan, seems to reflect motivation. It is known that self-efficacy interacts with motivation and behavior [16]. The residents in the simulation group felt that their behaviour improved more than that of the residents in the lecture group, as shown by the comments that they were motivated to learn more. Undoubtedly, they had other behaviour or awareness improvements that we were unable to detect in this study.

## Program evaluation

In terms of the difficulty of cases, only Case.1 was evaluated as being more difficult by the simulation group compared to the lecture group. However, there was no difference in the stress resulting from each training session; hence, case difficulty did not seem to be a problem for the trainees. They also expected that the simulation would relieve more stress in clinical situations than the lecture. McMillan et al. [17] have already reported that simulation training relieved PGY-I residents’ anxiety; hence, the PGY-I residents who participated in this simulation-based training were expected to feel less stress at clinical sites.

The residents were satisfied with the time spent in simulation-based learning and lectures during the orientation period. This is thought to have been derived from their anxiety to join clinical practice and their desire to rehearse clinical practice before starting it. In general, they will have similar experiences during simulation training in the future. On the other hand, lectures are inferior to simulation-based learning in terms of the depiction of reality. Therefore, conducting a simulation-based learning program for residents before starting clinical training might resolve the residents’ anxiety, satisfy their desire for practice, and be more meaningful than lectures.

Compared to the trainees in the lecture group, those in the simulation group more strongly felt that the PGY-II residents were appropriate teaching staff. In the free response questionnaire, they felt an affinity toward the PGY-II residents and the PGY-II residents had more empathy for them, having been in their shoes. Although it is a mainstream practice to have senior residents as teachers of other junior residents in Japanese clinical sites, there have been few studies which have focused on PAL using simulation-based education. In addition, there are general concerns that PGY-II residents may have insufficient knowledge and incorrectly teach topics. This negative aspect of PAL seemed more pronounced in simulation education than in lectures where tutors only teach prepared content. However, our program comprising only three hours of simulation training conducted by PGY-II residents revealed an equivalent effect on knowledge acquisition and a number of positive outcomes in the residents’ self-evaluation of competence and behaviour-change and program evaluation over the traditional lecture-style training conducted by the same residents. There is a positive correlation between learning time and learning effect in simulation-based education [18]. In conclusion, it is suggested that simulation-based education conducted by PGY-II residents was more effective for PGY-I residents than traditional lectures.

Often, the staff who provide some of the special simulation-based trainings for the acquisition of specific medical skills are experts who have already received special training [13]. It is difficult for all hospitals to prepare such teaching staff. While PAL can potentially resolve the problem posed by the shortage of simulation-based learning tutors, from an ethical perspective, we must not force PGY-II residents to become tutors without demonstrating the associated advantages to them.

It is noted that our study has a few limitations. First, this study only suggests that PGY-II residents are more appropriate teaching staff for simulations than for lectures based on the comparison of data and free response questionnaires. Graham et al. [19] previously suggested that the use of PAL techniques and medical students to teach physical examinations is a comparable level of training to that of training by experts; a comparison study should be performed on simulation-based learning by PGY-II residents’ PAL and experts teaching from the perspective of educational effects on trainees. Second, since we evaluated only the educational effects on PGY-I residents, it is necessary to consider the educational effects on PGY-II residents when they teach simulation-based learning. Third, since this study was conducted in a single facility and the questionnaires were limited in terms of validity in the course of their creation, this study should be considered a preliminary study, and further studies should be conducted in more facilities. Fourth, we examined only short-term educational effects and did not investigate the outcome in clinical situations. Currently, we are analysing data on PGY-I residents after they began clinical practice, focusing on the longer-term educational effects of this program. In addition, we evaluated their behaviour-change subjectively, rather than objectively, using questionnaires during the simulation and lecture. Future studies should examine the topic both subjectively and objectively.

## Conclusions

Simulation-based learning and PAL are known to be effective educational methods. This study clarifies that a combination of these methods led by PGY-II residents, rather than specialists, can potentially better improve postgraduate education for PGY-I residents compared to a combination of lecture and PAL.

## Abbreviations

PGY: Postgraduate-year
PAL: Peer-assisted learning.
SD: Standard deviation.
IQR: Interquartile range.
